# Surfactant–Polymer Composition for Selective Water Shut-Off in Production Wells

**DOI:** 10.3390/gels10020117

**Published:** 2024-02-01

**Authors:** Lyubov Magadova, Mikhail Silin, Vladimir Gubanov, Svetlana Aksenova

**Affiliations:** Department of Technology of Chemical Substances for the Oil and Gas Industry of Gubkin University, World-Class Research Center «Efficient Development of the Global Liquid Hydrocarbon Reserves», National University of Oil and Gas (Gubkin University), 119991 Moscow, Russia; silin.m@gubkin.ru (M.S.); gubanowww@gmail.com (V.G.)

**Keywords:** selective water shut-off, oil reservoirs, hydrolyzed polyacrylonitrile, viscosity, polymer gels, gel-forming composition, gel structure, adsorption, crosslinking agent, surfactant, filtrate, mineralized water

## Abstract

Today, a significant part of production wells’ stock has a high water cut percentage of 90% and above. Obviously, for this reason, the need to develop new and improved existing technologies for water shut-off in wells increases every year. Physico-chemical methods of water shut-off are based on the application of special reagents and compositions that plug the pathways of water inflow to the well. Depending on the mechanism and specific features of water barrier formation, isolation methods are divided into selective and non-selective. This article investigates the possibility of using hydrolyzed polyacrylonitrile as a gel-forming and precipitation-forming reagent for water shut-off technologies in production wells. A surfactant–polymer composition for the isolation of water inflow in production wells in objects with high salinity in formation water, possessing physical and chemical selectivity and providing permeability reduction only in water-saturated intervals, is proposed. The developed composition is the invert emulsion, which makes it possible to carry out treatment at a distance from the well and solve the problem of possible premature gel formation directly in the wellbore. The lowest effective concentration of HPAN in an aqueous solution for use as a gel-forming and sedimentation reagent was determined experimentally (5.0 wt% and more). The interaction of the polymer solution with a chromium crosslinker allows obtaining structured gels in the whole volume of the system. The structure of the gels was evaluated using the Sydansk classifier with the assignment of a letter code from A to J. It was experimentally proved that the structure of the obtained gels depends on the temperature and content of the crosslinking agent in the system; the more crosslinking agent in the composition of the system, the stronger the structure of the resulting gel. The optimal ratio of polymer and crosslinking agent to obtain a strong gel was obtained, which amounted to 5:1 by weight of dry polymer powder. For the HPAN concentration of 5 wt% according to the Sydansk classifier, the gel structure had the code “H”—slightly deformable non-flowing gel. The dependence of the volume of gel sediment obtained because of the interaction with mineralized water on the polymer concentration was studied. It was proved that an increase in the concentration of hydrolyzed polyacrylonitrile in the solution, as well as an increase in the concentration of calcium ions in mineralized water, leads to a larger volume of the resulting gel or precipitate and to the strengthening of the gel structure. The results of rheological studies of the developed composition, as well as experiments on thermal stability, are presented. The results of filtration tests on bulk reservoir models demonstrated the selectivity of the developed composition. The obtained value of the residual resistance factor for the oil-saturated low-permeability model was 1.49 units; the value of the residual resistance factor for the water-saturated high-permeability model was 18.04 units. The ratio of the obtained values of the residual resistance factor, equal to 0.08 (much less than 1), can characterize the developed composition as a selective material for water shut-off in producing wells. Existing technologies for water shut-off based on HPAN do not allow for making a treatment at a distance from the well and require the use of technological solutions to prevent premature gel sedimentation in the well. The developed composition makes it possible to solve the problem of premature gelation. In addition, the composition can form a blocking screen in highly permeable water-saturated zones. The development can be useful for deposits with difficult conditions (high mineralization in reservoir waters, boreholes with a horizontal end, elevated temperatures up to 80 °C).

## 1. Introduction

High rates of oil production by waterflooding at oil fields and the geological and geophysical features of the structure of productive formations lead to the intensive and rapid watering of produced wells long before reaching a potentially possible level of oil production. The main reasons for the watering of producing wells are [1]:

- A breach of tightness in the production string;
- Water inflow through leaky borehole space from above- or below-lying aquifers;
- An inflow of contour or injected water;
- Water inflow through fractures;
- Anthropogenic factors (for example, acid treatments and hydraulic fracturing).

Geologic causes of the premature watering of oil wells also include oil–water contact (OWC) movement, water cone formation, and injection water breakthrough through highly permeable interbeds. Since the inflow area (drainage zone) is large and the upward velocity of OWC is low, its rise can occur at very low vertical permeability. Cone formation occurs in vertical wells where the OWC is near lower perforations in formations with relatively high vertical permeability.

Technological methods of well water cut prevention include:

- The qualitative and reliable separation of productive formations during well construction;
- The selection of optimal underbalance on the reservoir during well development and operation;
- The selection of an optimal speed for oil displacement front advancement with water, corresponding to the speed of capillary water impregnation of reservoir rocks;
- Selection of the well operation mode (fluid withdrawal), taking into account the low mobility of the water–oil contact surface within the radius of a deep depression funnel;
- Equalization of the injectivity profile of injection wells by the application of fine fillers and polymer compositions;
- Changing the rheological properties of injected water by applying polymer flooding;
- Forced fluid withdrawal from the reservoir.

There are many classifications of water shut-off methods. In a general sense, these methods are divided into mechanical and physico-chemical methods. The former include running an additional production string, installing corrugated, retrievable, and polymer patches, and using a two-packer system. Various technical devices and technological methods have been proposed to prevent the formation of cone watering and extend the life of wells (e.g., joint and separate operation) [1,2,3].

Physico-chemical methods of water shut-off are based on the application of special reagents and compositions that plug the pathways of water inflow to the well. Depending on the mechanism and specific features of water barrier formation, isolation methods are divided into selective and non-selective. According to the type of action, there are curing, gel-forming, and sediment-forming selective reagents, as well as water repellents and foam systems. Gel-forming and sedimentation technologies for water shut-off are of particular interest at present.

In addition, for horizontal wells, due to, as a rule, insufficient information about the sources of watering, the large length of the productive interval (which requires large volumes of injected working reagent solutions), and the likelihood of complications during the treatment process due to the formation of clogging substances already in the wellbore, the most preferable is the use of selective materials and buffer rims.

Several requirements are imposed on selective compositions for water shut-off in production wells [1]:

- Selectivity of the compositions’ impact on the formation depending on fluid saturation (oil or water);
- Good selective filterability of compositions into zones with high water saturation, allowing the creation of water shut-off screens in the desired direction and to a sufficient depth;
- Adjustable timing of an isolating barrier build-up of the watered section of the formation according to the degree and duration of blockage;
- The ability to regulate technological properties in well conditions;
- Economic accessibility and non-scarcity;
- Environmental safety.

It is important to note that there are no completely selective materials. The selective sealant should be much more effective at blocking high-permeability, water-saturated areas with minimal impact on low-permeability, oil-saturated areas. 

One of the promising reagents from the perspective of use in technologies for selective water shut-off in production wells is hydrolyzed polyacrylonitrile (HPAN) and compositions based on it.

**Figure 1:**
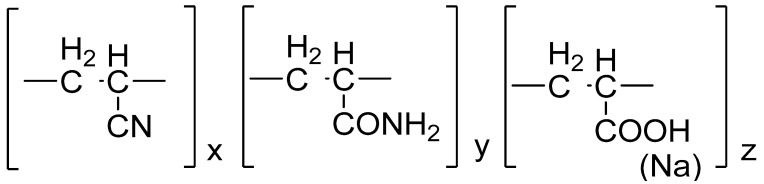
Structural formula of the HPAN molecule.

HPAN is a water-soluble polymer containing in its macromolecule chains of acrylonitrile, acrylamide, and sodium acrylate in a ratio of 1:1:8 (Figure 1). 

In industry, HPAN can be obtained by polymerization of acrylic acid nitrile followed by hydrolysis with sodium hydroxide, as well as alkaline hydrolysis.

**Figure 2:**
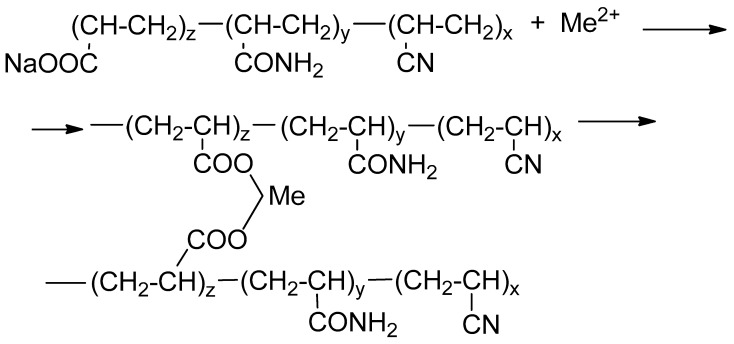
Mechanism of formation of a gel sediment during the interaction of HPAN with cations of polyvalent metals (Me).

When a solution of HPAN interacts with ions of polyvalent metals such as Ca^2+^, Mg^2+^, Fe^2+^, the polymer instantly coagulates to form a sediment or elastic gel of varying degrees of strength (Figure 2).

Among the electrolytes that cause coagulation of HPAN with the formation of a sediment or elastic gel, one can mainly note calcium, magnesium, iron (II), barium chlorides, and potassium permanganate. Coagulation of HPAN with hydrochloric acid is also possible. Several works are devoted to assessing the influence of sedimentators on the coagulation of HPAN [4,5,6,7,8,9,10,11]. In [4], the authors studied the mechanisms affecting the colmatizing properties of polymer liquids. In the laboratory, studies were carried out on a solution of HPAN with solutions of salts of polyvalent metals. The interaction of CaCl_2_, widely used in the oil industry for the precipitation of gels, as well as MgCl_2_ and FeCl_3_ with 5 wt% HPAN solution was compared. A significant difference was found in the concentrations of these salts, causing visible gelation in the solution and its precipitation. Thus, the beginning of visible coagulation of HPAN with a concentration of 5 wt% solution occurs when more than 200 mg/L of CaCl_2_ and more than 2000 mg/L of MgCl_2_ are injected into the solution, and FeCl_3_ causes visible coagulation when 6 mg/L is injected into the solution. 

It was noted that the compositions containing partially hydrolyzed polyacrylonitrile and a crosslinking agent (aluminum chloride) under the conditions of Kogalym and Langepas oil fields are capable of forming stable gels with a high water shut-off potential. The porosity of the reservoir decreased by 50 times or more [7].

A new composition is presented in the work in [8] Местo для ввoда текста. based on hydrolyzed polyacrylonitrile COM-C for limiting water shut-off in wells. COM-C is a new gel-forming compound capable of forming a durable shut-off screen in highly permeable areas. Hydrogels are formed when the composition interacts with hydrochloric acid.

Polymers based on acrylic acids contain carboxyl ionogenic groups, which facilitate their dissolution in water, interaction with electrolytes, and the formation of a durable insulating screen. The chromium (III) cation is bonded to two carboxylate groups of the polymer and to two water molecules. In this case, carboxylate groups act as bidentate ligands [12]. Thus, carboxylate groups of HPAN play a crucial role in gel formation.

When interacting with oil, coagulation of HPAN does not occur. This mechanism allows the use of HPAN in water shut-off technologies for selective isolation of watered layers [13,14,15,16,17]. 

**Figure 3:**
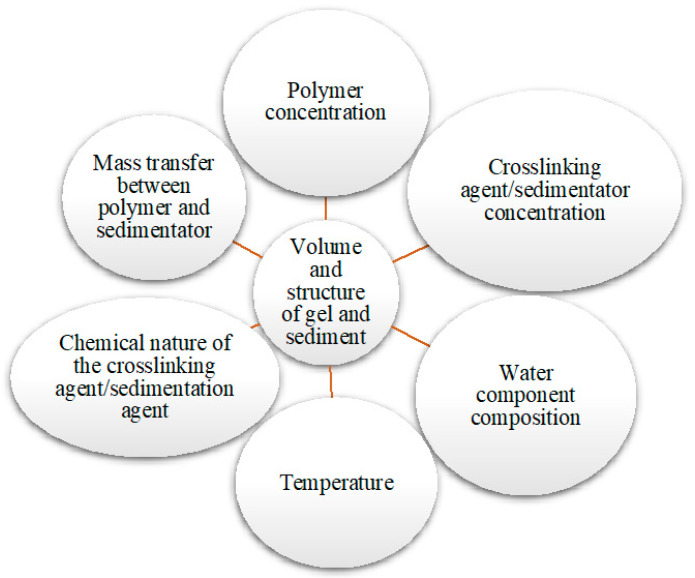
Factors affecting the properties of HPAN gel and sediment.

The properties of the gel or sediment formed during the interaction of HPAN with various sedimentators are influenced by many factors, from the concentration of the polymer and the nature of the sedimentator to mass transfer between the polymer and the sedimentator (Figure 3).

Based on the available data on the use of such technologies in production wells, it becomes obvious that the efficiency of treatment is directly affected by the total mineralization of formation waters, in particular, the total content of calcium and magnesium cations. Therefore, compositions based on HPAN are, as a rule, used at sites with a total mineralization of at least 50 g/L. Technologies include mandatory pre-flushing of the well (to avoid premature gel sedimentation of HPAN) and injection of a polymer solution and a calcium chloride solution separated by a buffer liquid.

The main disadvantage of the known technologies is that the formation of an insulating screen in the form of a gel sediment when the resulting composition interacts with formation water containing polyvalent metal ions occurs almost instantly. Thus, premature sedimentation and the formation of gel sediment in the wellbore are possible, which complicates the technology of water shut-off treatment [13,15,18,19,20]. 

Existing technologies for water shut-off based on HPAN do not allow for treatment at a distance from the well and require the use of technological solutions to prevent premature gel sedimentation in the well.

Therefore, the purpose of this work is to develop a gel-forming composition in the form of an invert emulsion for isolating water inflows in production wells at sites with high mineralization of formation waters, which has physical and chemical selectivity and ensures a reduction in the permeability of only water-saturated intervals. 

The article presents the results of laboratory studies on the selection of the concentration of HPAN and the crosslinking agent. The influence of various factors on the coagulation of HPAN was studied. Rheological studies of the developed composition and studies of emulsions for thermal stability were carried out. Filtration tests of the developed composition were carried out on bulk reservoir models using real formation fluids.

## 2. Results and Discussion

The developed surfactant–polymer composition for selective water shut-off in production wells is an invert (water-in-oil) emulsion, the dispersion medium of which is represented by a hydrocarbon surfactant solution, and the dispersed phase is a solution of HPAN with a chromium crosslinker. The following sections of this work present the results of studies of the developed composition.

**Figure 4:**
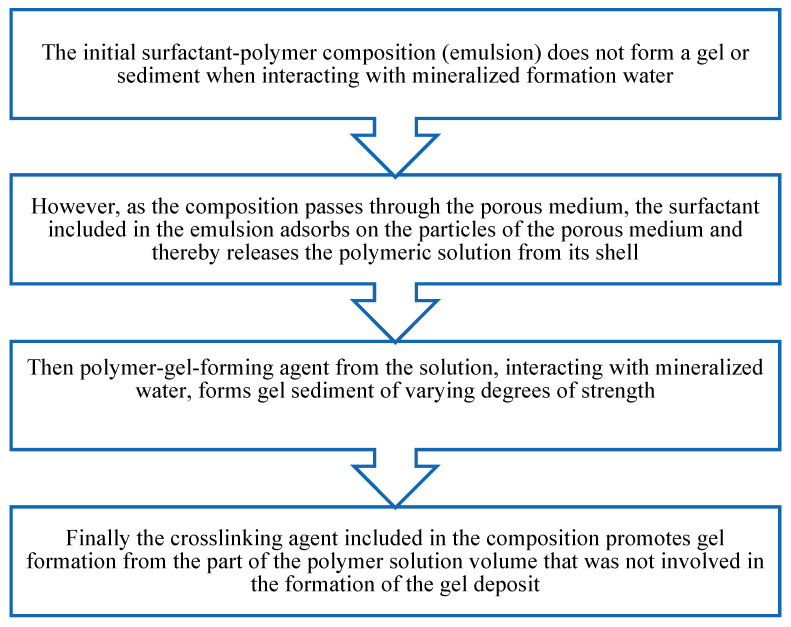
Mechanism of action of the surfactant–polymer composition.

The mechanism of action of the developed surfactant–polymer composition can be represented by the diagram below (Figure 4). Initially, the inverse emulsion is a stable system and does not form a gel or sediment when interacting with formation water. As the composition moves through the pore space of the formation, the emulsion is destroyed, and the hydrocarbon-soluble surfactant is adsorbed on the surface of the porous medium. Thus, the polymer solution with the crosslinking agent (HPAN and CA) is freed from the surfactant shell. Upon contact with formation mineralized water, HPAN coagulates to form sediment. The remaining part of the polymer, when interacting with the crosslinking agent, forms a gel. 

First, it is necessary to determine the optimal concentration of HPAN and crosslinking agent. 

### 2.1. Selection of the Conqcentration of Hydrolyzed Polyacrylonitrile

The most important property of any composition for water shut-off in production wells is its rheological characteristics. The developing composition must be sufficiently pumpable under downhole conditions. With increasing concentration of the polymer in the solution, the viscosity naturally increases. The systems are non-Newtonian fluids with pseudoplastic flow behavior, which is typical for polymer systems. Based on the Russian experience in using HPAN, it can be noted that the polymer concentration usually does not exceed 10 wt%. The use of a polymer at a concentration of 10 wt% increases the viscosity of the composition so much that it cannot be pumped into the formation using standard equipment, and also increases the load on pumping equipment when pumping such a viscous composition. Therefore, an increase in the concentration of HPAN by more than 10 wt% is impractical.

**Figure 5:**
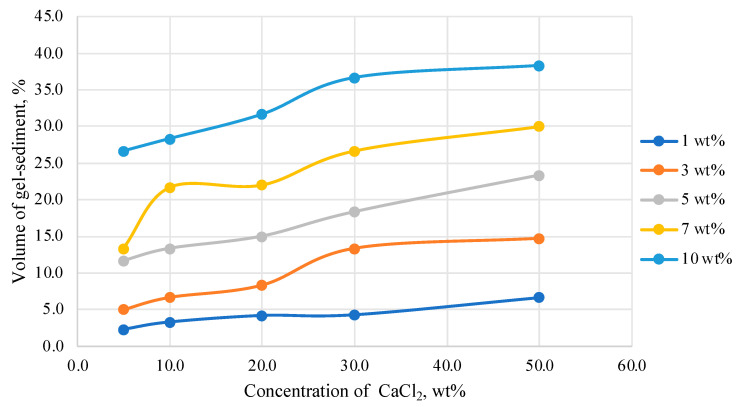
Dependence of the volume of gel sediment on the concentration of calcium chloride in solution at 25 °C.

The dependence of the volume of the HPAN gel sediment on the concentration of calcium chloride in the solution is shown in Figure 5.

From the graph data, the volume of the HPAN gel sediment directly depends on the concentration of the polymer in the solution; the higher the concentration of HPAN, the greater the volume of the resulting gel or sediment. It is very important to obtain the maximum volume of gel sediment in the system. It is assumed that the greater the volume and strength of the resulting gel sediment, the better the effect of work to limit water inflow. 

And, of course, when operating wells, it is necessary to use technologically advanced and economically accessible compositions. For example, if 25–30% of the volume of gel or sediment is obtained from 100% of the composition, then this is not economically feasible. 

The smallest volume of sediment is observed in the system with an HPAN concentration of 1.0 wt%. The mass formed when a polymer solution is mixed with calcium chloride is a loose sediment that cannot create a water shut-off barrier necessary when carrying out work for water shut-off (Table 1).

However, even at relatively high polymer concentrations (10.0 wt%), the volume of the gel sediment does not exceed 40% of the total volume of the system. In technologies of water shut-off in production wells, this may not be enough for effective work. Thus, there is an interest to test the possibility of increasing the volume of the gel sediment formed by the polymer when it interacts with mineralized water.

**Figure 6:**
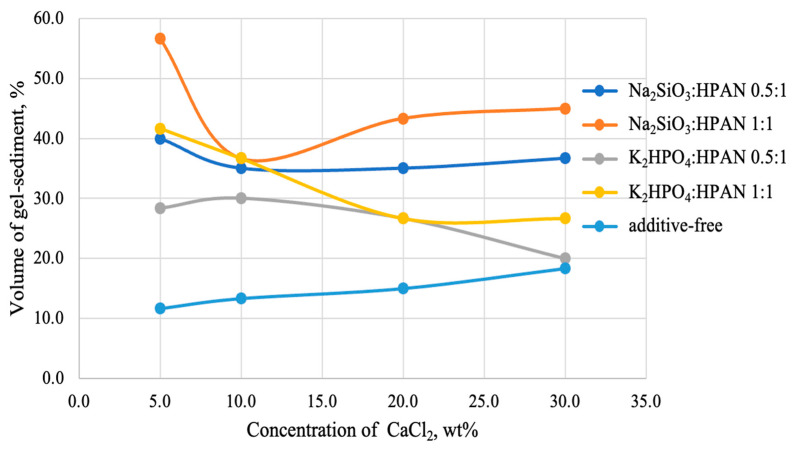
Dependence of the volume of gel sediment on the concentration of calcium chloride in the solution (using the example of a 5 wt% HPAN solution) at 25 °C.

After adding salts to the polymer solution (the amount of the additive was introduced based on the dry polymer powder) and mixing the system with a solution of calcium chloride, the volume of the gel sediment actually increases (Figure 6), but, unfortunately, to an insufficient extent (no more than 55% of total volume of the system).

The presence of carboxyl groups in the HPAN molecule makes it possible to obtain gels throughout the entire volume by reacting the polymer with chromium crosslinkers. An example of such a system is the interaction of a solution of HPAN with chromium (III) acetate. It is assumed that during the formation of a three-dimensional gel structure, the Cr^3+^ cation preferentially binds with two carboxyl groups of neighboring HPAN macromolecules and two water molecules. A similar process is observed during the interaction of another polymer of the acrylic series—polyacrylamide (PA)—which also contains carboxyl groups in its macromolecule [12,21,22]. 

By varying the ratio of polymer and crosslinking agent, it is possible to control the gelation time, as well as the structure of the resulting gels. Therefore, systems based on PA and chromium acetate have successfully found their application in technologies for water shut-off and leveling the injectivity profile [23,24,25,26,27,28,29]. 

The ability of HPAN to form structured gels with chromium (III) acetate makes it possible to obtain an additional volume of plugging mass to form a more durable insulating screen after the initial generation of a gel sediment in contact with reservoir water. 

### 2.2. Selection of Polymer: Crosslinking Agent Ratio

Polymer concentrations ranging from 3 wt% to 7 wt% were chosen to obtain crosslinked polymer systems throughout. The gelation process was observed at 25 °C and 70 °C to form a crosslinked three-dimensional structure. The research results are presented in Table 2.

It is important to note that gel formation occurred in the entire volume of the system (100%) in all samples where the polymer concentration was 5.0 wt%. or more. The structure of the resulting gels depends on the temperature and content of CA in the system; the more chromium acetate in the system, the stronger the structure of the resulting gel. Based on the results of observing the samples for 30 days, the structure of the formed gels did not change, and no release of water from the gels—syneresis—was observed. During the study, all samples were stored in sealed containers with screw caps at 25 °C and atmospheric pressure for 30 days. 

In samples with a polymer content of less than 5.0 wt%, the formation of a structured gel under experimental conditions did not occur within 24 h. To obtain strong gels with low concentrations of HPAN, it is necessary to increase the content of the crosslinking agent in the system (which may not be economically viable) or increase the gel holding time to more than 24 h (which is undesirable for well operations).

Thus, the optimal concentrations of the polymer and crosslinking agent were selected for preparing the dispersed phase of the emulsion that is being developed—at least 5 wt%. for HPAN and a polymer:crosslinking agent ratio = 5:1 (based on dry polymer powder) to obtain the most durable structured gel. For the HPAN concentration of 5 wt%, according to the Sydansk classifier, the gel structure had the code “H”—slightly deformable non-flowing gel. The “tongue” of the gel was not fixed; the gel was practically non-flowing. The research results provided below are given for compositions with a polymer concentration of 5 wt%.

### 2.3. Determination of Aggregative Stability and Thermal Stability of an Emulsion

The stability of emulsions is affected by temperature, pH, salinity, viscosity of the dispersion medium, and other factors. To increase the stability of emulsions, various methods are used (introducing stabilizing reagents, increasing the viscosity of the dispersion medium, changing the phase ratio). 

Among the main factors influencing the stability of the developed system are the concentration of the surfactant stabilizer, the “dispersed phase: dispersion medium” ratio, and temperature.

Based on the results of preliminary laboratory studies, the optimal concentration of the surfactant stabilizer in the composition was determined. It amounted to 15–20% of the total volume of the hydrocarbon phase of the emulsion. The surfactant concentration was determined based on tests for the interaction of emulsion samples with mineralized water (produced water or calcium chloride solution), followed by an assessment of the system for the presence of a gel or HPAN sediment, as well as thermal stability tests of emulsion samples. An insufficient amount of surfactant stabilizer in the system also leads to low values of aggregation stability and thermal stability of the emulsion. If the emulsion is not stable enough (surfactant stabilizer content in the hydrocarbon phase of the emulsion is less than 15%), the polymer, freed from the surfactant stabilizer shell, when mixed with the formation water, instantly forms a coagulation film upon contact.

The thermal stability of the emulsion composition is the most important indicator of its applicability. This indicator characterizes the temperature limits at which the composition can be used, and shows the possible sedimentation, release of gases, and the release of phases that make up the emulsion, which indicates the unsuitability of the composition.

In this case, the composition must be sufficiently stable for successful injection into the well. Therefore, the samples were tested for thermal stability for 8 h. If the emulsion is not stable enough, it can break down in the wellbore and cause premature coagulation of HPAN upon contact with saline water. The emulsion must break down as it passes into the pore space of the formation, and HPAN must interact with the formation water after adsorption of the surfactant into the porous media.

**Figure 7:**
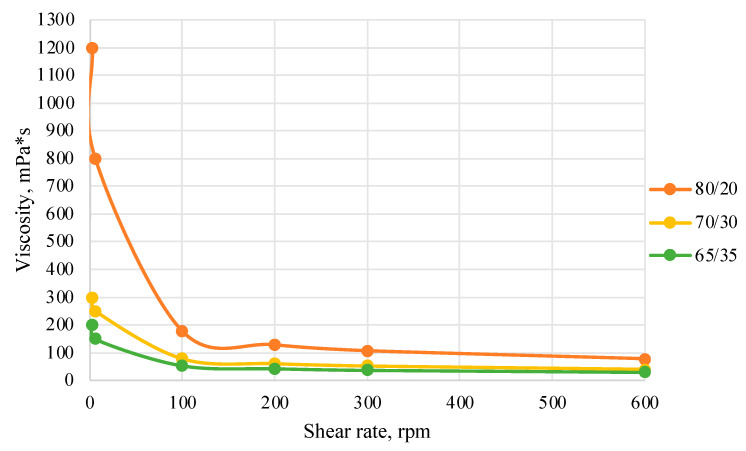
Rheological parameters of emulsions at 25 °C.

As part of testing the compositions for thermal stability, samples of emulsions with different ratios of dispersed phase and dispersion medium were placed in graduated tubes with screw caps and thermostated in a heating cabinet at temperatures of 25 °C, 40 °C, and 70 °C for 8 h. All samples were checked every hour and preliminary results were recorded. After the exposure time had expired, samples were taken for examination under a microscope (magnification factor—40×). For each sample, the amount of hydrocarbon phase separated from above was also recorded. The results are presented in Table 3. The rheological parameters of the emulsion samples are presented in Figure 7. As the content of the dispersed phase in the system increases, the viscosity of the emulsion increases.

The most stable is an emulsion with a “dispersed phase:dispersion medium” ratio of 80/20.

### 2.4. Interaction of the Composition with Oil and Formation Mineralized Water

To assess the compatibility of the developed composition with oil, a sample of freshly prepared emulsion was mixed with oil in a ratio of 1:1 by volume and stirred using a top-drive paddle mixer at a speed of 300–350 rpm for 5 min. Next, the sample was poured into a sealed vessel with a screw cap.

When the water shut-off composition interacts with oil, the formation of flakes, sediments, emulsions, or the release of a new phase should not occur.

The selectivity of invert emulsions lies in the fact that upon contact with oil, the emulsion is diluted and its viscosity is significantly reduced compared to the original. When interacting with water, the emulsion gains viscosity, which makes it possible to create an insulating screen for use in water shut-off technologies. The proposed surfactant–polymer composition does not form any sediments with oil (Table 4). The viscosity of the “surfactant–polymer composition:oil” system decreases by 10–15 times relative to the initial viscosity of the composition. 

### 2.5. Compatibility of the Composition with Process Fluids

To assess the compatibility of the developed composition with displacement fluids, a sample of freshly prepared emulsion was mixed with the displacement fluid in a ratio of 1:1 by volume and stirred using an overhead stirrer at a speed of 300–350 rpm for 5 min. The samples were then placed in sealed containers with screw caps and thermostated in a drying cabinet at 25 °C for 24 h and 70 °C for 8 h. When the water shut-off composition interacts with the displacing fluids, there should be no formation of flakes, sediments, emulsions, or separation of a new phase.

The proposed surfactant–polymer composition does not form any sedimentation with the liquids under consideration. The system is completely divided into the “top layer”—the surfactant–polymer composition—and the “bottom layer”—the displacement fluid.

### 2.6. Laboratory Assessment of the Action Mechanism of the Composition: Filtration Studies

**Figure 8:**
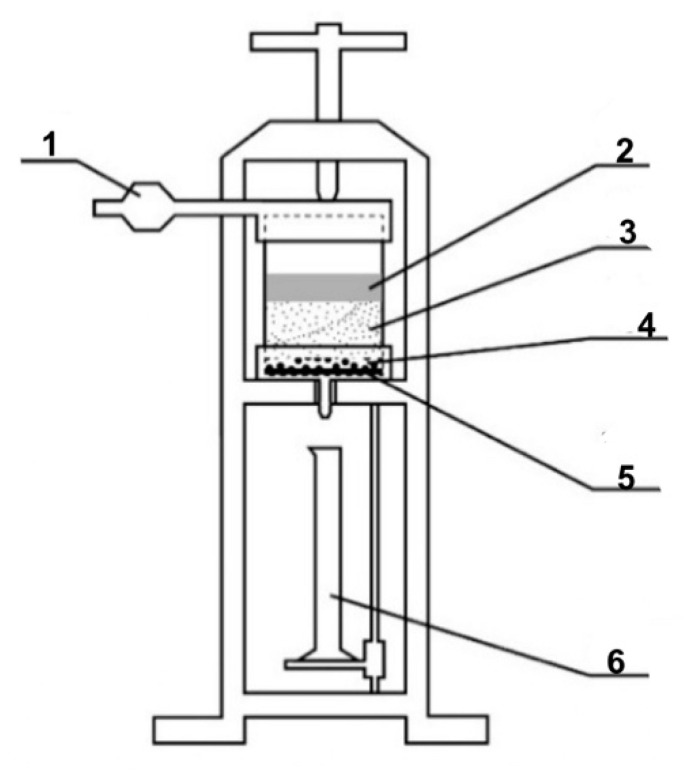
Filling diagram of the fluid loss cell installation: 1—injection line; 2—filtered solution; 3—sand placement; 4—gravel placement; 5—metal mesh (filter); 6—measuring cylinder.

At the first stage of research, the action mechanism of the developed composition was assessed using a fluid loss cell for drilling fluids. A thin layer of gravel filter was poured into a clean and dry cell of the Fann LPLT model 300 fluid loss cell to prevent sand from passing through the mesh holes, then quartz sand of BC-030-B grade, fraction 0.1–0.4 mm, was poured (Figure 8).

Freshly prepared emulsion was poured into the cell from above. The quartz sand:emulsion ratio was 2:1 (by volume). Excess pressure (about 2 atm) was supplied to the fluid loss cell. The filtrate coming out of the sand pack was a brown liquid.

After the filtering process was completed, the device cell was disassembled, visually assessing the condition of the sand pack. By the even color of the sand pack, one can indirectly judge the uniform passage of the emulsion composition.

Then, mineralized water was gradually poured into the resulting filtrate while stirring (the “filtrate:mineralized water” ratio was 1:1 by volume). At the end of mixing, the state of the system was visually assessed.

**Figure 9:**
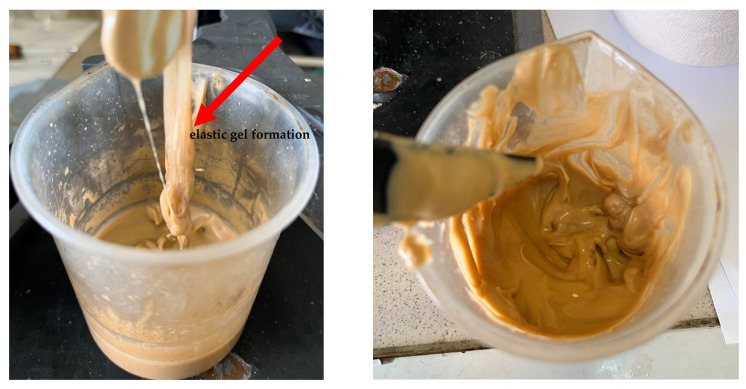
Formation of gel sediment from the filtrate of the working solution and reservoir water.

When the filtrate of the working solution of the composition is mixed with mineralized water, an elastic gel-like mass is formed, and coagulation of HPAN occurs when the polymer interacts with salts of polyvalent metals contained in the formation water (Figure 9). There is a general increase in the viscosity of the entire system. Keeping the system for the next 24 h allows one to form an additional volume of gel due to the presence of a crosslinking agent in the composition.

Based on the results of the experiments, it can be concluded that in the process of filtering the emulsion through a dry sand pack, the process of destruction of the emulsion occurred, in addition to the adsorption of surfactants on sand particles, and as the composition passed through the filling, a polymer solution was released.

To assess the effectiveness of the technology for water shut-off using the developed surfactant–polymer composition as part of filtration studies, physical modeling of the process of injection of the composition into the bottomhole zone of the formation was carried out using bulk models of the formation with different permeability (a water-saturated model with residual oil for modeling washed intervals with high permeability, as well as an oil-saturated model with residual water for modeling unwashed areas with lower permeability). 

Testing of the plugging properties of the surfactant–polymer composition (emulsion with HPAN 5 wt% and HPAN:CA ratio 5:1) on a water-saturated model was carried out on a bulk reservoir model with permeability with 100% water saturation of 1.17 μm^2^, length 22.1 cm, and diameter 2.6 cm. Filtration was carried out at the reservoir temperature of 40 °C. Produced water from one of the fields in the Samara region with a dynamic viscosity value of 1.32 mPa·s and a density of 1156.2 kg/m^3^ under reservoir conditions was used as a model of the displacing agent.

The model was saturated with formation water and retained its pore volume and porosity. In the process of water filtration, the coefficient of initial water permeability is achieved at the experimental temperature $k_{pr0}^{w}$.

At the next stage, 1 V_por_ of a working solution of the emulsion was injected into the model without back pressure at flow rate FlR = 0.5 cm^3^/min in the “well-formation” direction in a maximum volume equal to the pore volume of the model, 1 V_por_, or until a specified maximum pressure drop was achieved. After holding the model for a specified time (1 h), formation water was pumped into it in the “formation-well” direction. Then, after holding for 15 h, formation water was again injected in the “formation-well” direction. The phase permeability for water k^w^_pr1_ was determined.

From the obtained values of the initial and final permeability coefficients, the residual resistance factor ((Equation (1)) was calculated:

(1) $$F_{RR}=\frac{k_{pr0}^{w}}{w_{pr1}^{w}}$$

where F_RR_ is residual resistance factor, units, $k_{pr0}^{w}$ is initial water permeability at the experimental temperature, and $w_{pr1}^{w}$ is phase permeability to water, μm^2^.

**Figure 10:**
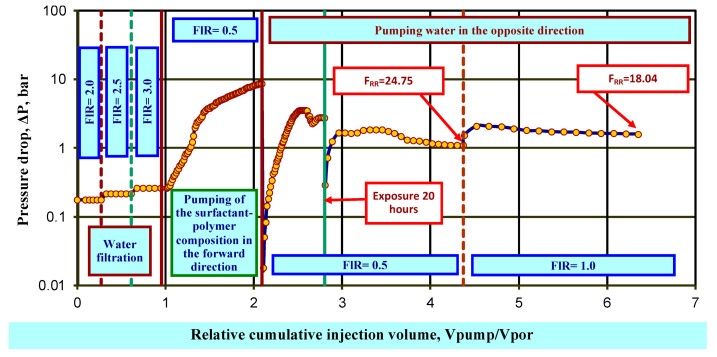
Dynamics of pressure drop during the sequential injection of the composition and formation water in the opposite direction after exposure into a water-saturated bulk model.

After filtration of formation water in the opposite direction, the residual resistance factor F_RR_ was 18.04 units. (Figure 10). 

Testing of the plugging properties of the surfactant–polymer composition on an oil-saturated model with residual water saturation was carried out on a bulk reservoir model with a water permeability of 0.427 μm^2^, a length of 22.2 cm, and a diameter of 2.6 cm. Filtration was carried out at a reservoir temperature of 40 °C.

The model was saturated with formation water, and its pore volume and porosity were determined. In the process of water filtration, the coefficient of initial water permeability at room temperature, $k_{pr0}^{w}$, was determined.

Next, residual water was created in the reservoir model by pumping oil from a field in the Samara region from a high-pressure vessel under a pressure of about 2.0 MPa into a vertically located formation model.

At the next stage, in the process of oil filtration in the “reservoir-well” direction, the phase permeability coefficient for oil was determined before the exposure of $w_{pr1}^{o}$ at the experimental temperature.

Then, a 1 V_por_ working solution of the emulsion gel sedimentation composition was injected into the bulk model without back pressure at flow rate FlR = 0.5 cm^3^/min in the “well-formation” direction in a maximum volume equal to the pore volume of the model, 1 V_por_. After holding the model for a specified time (1 h), oil was injected into it in the “formation-well” direction. Then, after holding for 48 h, oil was again filtered in the “formation-well” direction. The phase permeability of oil, $w_{pr2}^{o}$, was determined.

The residual resistance factor F_RR_ (units) for an oil-saturated low-permeability model with residual water saturation is determined by the ratio of the oil permeability before injection to the oil permeability after injection of the test composition.

**Figure 11:**
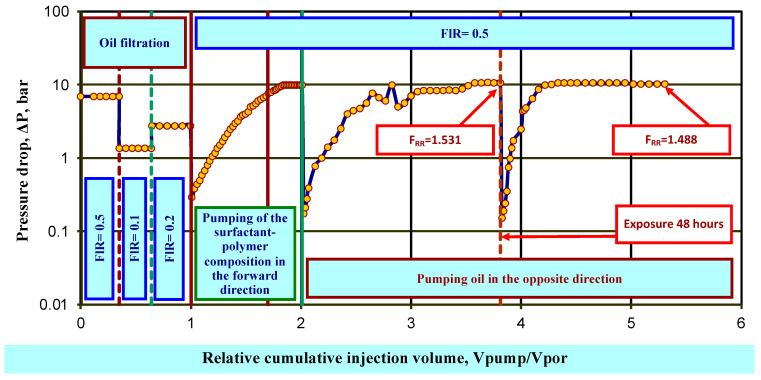
Dynamics of pressure drop during the sequential injection of the composition and formation oil in the opposite direction after exposure into an oil-saturated bulk model.

After oil filtration in the reverse direction, the residual resistance factor F_RR_ was 1.49 units (Figure 11).

The dynamics of pressure drop during the process of sequential injection of the composition and formation oil in the opposite direction after holding into a bulk oil-saturated model with residual water are shown in Figure 11. The obtained value of the residual resistance factor for the oil-saturated low-permeability model is much lower than the value obtained for the water-saturated high-permeability model. The ratio of the obtained values of the residual resistance factor equal to 0.08 (significantly less than 1) can characterize the surfactant–polymer composition as a selective material for water shut-off in production wells.

## 3. Conclusions

The work describes the stages of development and laboratory research of a surfactant–polymer composition for selective water shut-off in production wells at sites with high salinity of formation waters. The structure of the macromolecule and the chemical properties of HPAN allow the polymer to be used as a gel-forming and sediment-forming reagent. Traditional HPAN-based technologies involve alternating injection of a polymer solution and formation water (or calcium chloride solutions), separated by buffer fluids. The developed composition is an invert emulsion, which makes it possible to carry out treatment at a distance from the well and solve the problem of possible premature gel sedimentation directly in the wellbore (which is especially important in complex objects, including horizontal wells).

As part of laboratory studies, the optimal polymer concentrations (4 wt% or more) and the amount of crosslinking agent (with a polymer:crosslinking agent ratio of 5:1 (calculated on dry polymer powder)) were selected; it is possible to obtain the most durable gel structure. To ensure the stability of the developed composition, the concentration of the emulsifier and the optimal ratio of the dispersed phase and the dispersion medium were selected. To prepare a stable invert emulsion, the optimal concentration of the surfactant emulsifier was selected; this concentration was 15–20% of the entire hydrocarbon phase of the system.

Laboratory tests of the developed surfactant–polymer composition demonstrate the absence of interaction of the composition with displacement fluids, as well as with mineralized formation water (before filtration of the composition in a porous medium).

It has been experimentally proven that the composition has not only physical, but also chemical selectivity, forming a gel sediment only in water-saturated, highly permeable zones. The obtained value of the residual resistance factor for the oil-saturated low-permeability model was 1.49 units, and the value of the residual resistance factor for the water-saturated high-permeability model was 18.04 units. The ratio of the values of the residual resistance factor obtained from the results of filtration tests on a water-saturated high-permeability model and an oil-saturated low-permeability model was 0.08. When the developed composition comes into contact with oil, the process of sedimentation and gelation does not occur.

The developed composition allows us to solve the problem of premature gelation. In addition, the composition can form a blocking screen in highly permeable water-saturated zones. The development may be useful for fields with difficult conditions (high salinity of formation waters, boreholes with a horizontal end, elevated formation temperatures up to 80 °C).

## 4. Materials and Methods

### 4.1. Materials

The objects of study are deposits with high salinity values of formation waters. 

In the experimental part of the work, HPAN was used. HPAN is a yellow-to-brown powder with a mass fraction of water of no more than 7 wt% (Zavod Orgsintez Oka, Dzerzhinsk, Russia).

Chromium acetate was chosen as a crosslinking agent—chromium (III) acetate (acetate of chromium (III)) grade A, a dark green liquid with a mass fraction of chromium (III) of at least 11.35 wt%. (Petrokhim, Belgorod, Russia)

To prepare and stabilize the emulsion, we used an oil-soluble surfactant—emulsifier HS (Khimeko-Servis, Moscow, Russia)

The hydrocarbon solvent can be represented by summer diesel fuel (GOST 305-2013 [30]) or commercial oil, for example, oil from one of the fields in the Samara region with a density of 758 kg/m^3^ and a dynamic viscosity of 4.076 mPa·s at 25 °C.

Produced water from one of the deposits in the Samara region with a density of 1165.5 kg/m^3^ and a dynamic viscosity of 1.23 mPa·s at 25 °C was chosen as a source of polyvalent metal ions for the preparation of gels and sediments. The chemical composition of formation water is shown in Table 5. An aqueous solution of calcium chloride with a concentration of 200 g/L was also prepared to simulate highly mineralized water.

### 4.2. Experimental Methods

#### 4.2.1. Rheological Study

The rheological parameters of the resulting systems were studied using a FANN 35 SA rotational viscometer (R1-B1-F1) at six shear rates (3, 6, 100, 200, 300, and 600 rpm) at 25 °C. The results were processed in accordance with the instructions for the equipment. 

#### 4.2.2. Evaluation of the Effect of Salts on Gel Sediment Volume

One of the possible ways to increase the volume of the gel sediment may be to add salts to the polymer solution, which forms a sedimentation when interacting with Ca^2+^ cations in the solution.

The following were chosen as the object of study: potassium hydrogen orthophosphate dihydrate (K_2_HPO_4_·2H_2_O), acid salt, crystalline hydrate of calcium hydrogen orthophosphate, obtained by crystallization from its aqueous solution. Dipotassium hydrogenphosphate is used in the food industry, as a complex fertilizer, and also as a component for the preparation of buffer solutions. The wide distribution of this salt in various industries makes it economically available, including as a reagent for water shut-off into a well.

When dipotassium hydrogenphosphate interacts with calcium chloride, an exchange reaction occurs with the formation of a calcium hydrogen orthophosphate sediment (Equation (2)):

K_2_HPO_4_ + CaCl_2_ → CaHPO_4_↓ + 2KCl (2)

Another possible additive could be sodium silicate (Na_2_SiO_3_), an aqueous solution of which is known as “liquid glass” and is widely used in the construction, paint and varnish, and oil industries. The composition based on sodium silicate and chlorides forms insoluble, impermeable silicate precipitates (metasilicate, pyrosilicate, orthosilicate), which have good variations in chemical and thermal stability (Equation (3)):

(3) $${\mathrm{Na}}_{2}\mathrm{SiO}3_{\;}+{\mathrm{CaCl}}_{2}\;\to \;{\mathrm{CaSiO}}_{3}↓+2\mathrm{NaCl}$$

Injection of aqueous salt solutions is usually carried out in alternation, using buffer liquids, to prevent premature sedimentation.

By interacting sodium silicate solution with chromium crosslinking agents, structured gels with adjustable gelation times can be obtained [31,32,33]. 

Sodium silicate (which comes in a wide range of forms with varying ratios of sodium oxide (Na_2_O) and silicon dioxide (SiO_2_)) reacts with many chemicals to form a “sol” or gel strong enough to prevent water from moving into the formation [31]. The authors highlight the low cost, controlled gelation time, and stability of compositions at high temperatures.

The objects of study in [32] were compositions based on sodium silicate, partially hydrolyzed polyacrylamide A345, chromium acetate, and dispersed filler—rice husks. In this composition, chromium acetate is both a crosslinking agent for PAA and a catalyst for the polymerization of silicate ions of sodium silicate. Adding rice husk to the hydrogel increases the residual resistance factor and ultimate shear stress, which provides a good waterproofing effect when testing cores and wells.

The work in [33] presents the results of laboratory studies of the rheological and filtration characteristics of the composition. As a result of mixing the composition components (sodium silicate and chromium acetate), it is possible to obtain gels with adjustable gelation time from several minutes to several days and varying strength under conditions of a wide range of reservoir temperatures. The concentration of sodium silicate and chromium acetate is selected taking into account the formation temperature and purpose of application. During the injection process, the composition has a low viscosity, and after the well is shut down, a gel forms in reservoir conditions, plugging the water inflow channels.

To assess the effect of the concentration of the polymer solution and precipitant (in this case, calcium chloride) on the volume of the gel sediment, aqueous solutions of HPAN were mixed with CaCl_2_ solutions using an IKA EUROSTAR overhead paddle mixer with a stirring speed of 250–300 rpm at 25 °C in a ratio of 1:1 by volume. Next, the resulting gel or sediment was filtered using a water jet pump on a Buchner funnel, measuring the volume of remaining water. Experiments were carried out at a temperature of 25 °C. Thus, the volume of the resulting gel or sediment can be simplified by the following Equation (4):

(4) $$V=\frac{\left(V_{t}-V_{w}\right)}{V_{t}}\times 100\%,$$

where V_t_ is the total volume of the system (mL) and V_w_ is water volume (mL). 

To evaluate the effect of additives (K_2_HPO_4_·2H_2_O and Na_2_SiO_3_) on the HPAN solution, the following experiments were carried out. First, the additives were mixed with the polymer solution using an overhead paddle mixer with a mixing speed of 250–300 rpm at 25 °C. The amount of additive was introduced in different proportions by weight of the dry polymer powder. After mixing, solutions containing HPAN and the additive were mixed with an aqueous solution of calcium chloride in a 1:1 ratio by volume. The resulting gel or sediment was then filtered using a water jet pump on a Buchner funnel, measuring the volume of water remaining. Finally, the volume of the resulting gel or sediment can be simplistically calculated using Equation (4). 

#### 4.2.3. Gel Structure Assessment

To prepare crosslinked polymer systems based on HPAN, a calculated amount of chromium acetate (CA) was introduced into freshly prepared polymer solutions. Mixing was carried out using an overhead paddle mixer at a speed of 250–300 rpm at 25 °C until a homogeneous solution was obtained. Various ratios of HPAN:CA (from 5:1 to 100:1) were chosen for the studies. All the samples were then placed in a drying cabinet and preheated to the experimental temperature to form a crosslinked three-dimensional structure. 

The structure of the gels during the gelation process was assessed by bottle test using the Sydansk classifier in the form of a letter code from A to J [34,35,36,37]. According to this method [38], strength codes of gels ranged from viscous solutions to low-deformation, immobile gels (Table 6). 

#### 4.2.4. Preparation of the Emulsion

The emulsion was prepared in laboratory conditions as follows. The calculated amount of fresh water was poured into a glass beaker, and then, while stirring with an overhead stirrer (rotation speed 300–500 rpm), HPAN powder was gradually introduced and stirring was continued until a homogeneous solution was obtained throughout the entire volume. Then, without stopping stirring, a crosslinking agent, chromium acetate, was added.

A hydrocarbon solvent was poured into another glass beaker; then, while stirring with an overhead stirrer, the calculated amount of HS emulsifier was gradually introduced into the solvent.

To obtain a reverse emulsion, a solution of the emulsifier in a hydrocarbon solvent was poured into a narrow glass beaker and placed under an overhead stirrer. The stirring mode was turned on and a polymer solution with a crosslinker was slowly introduced dropwise into the surfactant solution from a separating funnel, gradually increasing the stirring speed to 1000 rpm.

After introducing the entire volume of the polymer solution, stirring was continued until a homogeneous emulsion was obtained.

#### 4.2.5. Filtration Studies

Filtration studies make it possible to predict the effect of the developed water inflow limitation technology and study the mechanisms of water shut-off screen development [37,39]. Filtration experiments are carried out using bulk or core reservoir models and real formation fluids under thermobaric conditions of the field.

In this case, filtration studies on the bulk reservoir model were carried out on the high-pressure and high-temperature filtration unit HP-CFS, which allows for a wide range of experiments to study the processes of fluid filtration through porous media under thermobaric conditions of the reservoir. 

The HP-CFS unit allows filtration experiments to be carried out at temperatures up to 150 °C and pressures up to 20.0 MPa. If necessary, a backpressure system is used, providing a maximum pressure level of 7.0 MPa. When working with core samples, the pressure testing can reach 50.0 MPa. 

The main functional parts of the unit are one or two temperature-controlled bulk reservoir models and core holders for experiments with core samples. Depending on the type of study, you can use a core holder for composite cores up to 30 cm in length, or a core holder for a single core sample.

First, it is necessary to prepare the bulk model as well as the formation fluids for study. 

## Figures and Tables

**Table 1:** Dependence of the coagulating effect of calcium chloride in relation to the HPAN solution on the concentration of calcium chloride at 25 °C.

HPAN Concentration, wt%	State of the “Polymer Solution: Calcium Chloride Solution” System at Different Concentrations of CaCl_2_, wt%				
	5.0	10.0	20.0	30.0	50.0
1.0	heterogeneous loose sediment	heterogeneous loose sediment	heterogeneous loose sediment	fine sediment	fine sediment
3.0	«G»	«G»	«G»	«H»	hard sediment
5.0	«G»	«G»	«G»	«H»	hard sediment
7.0	«G»	«G»	«G»	hard sediment	hard sediment
10.0	«G»	«G»	«G»	hard sediment	hard sediment

**Table 2:** Interaction of aqueous solutions of HPAN with chromium acetate (25 °C and 70 °C).

N°	Polymer Concentration, wt%	HPAN:CA Ratio	System Descripatizon
1	3.0	5:1	“A”—the viscosity of the system is comparable to the viscosity of the original solution
2	3.0	10:1	“A”—the viscosity of the system is comparable to the viscosity of the original solution
3	5.0	5:1	“H”—Slightly deformable non flowing gel; the “tongue” is not fixed—the gel is practically non-flowing
4	5.0	10:1	“G”—Moderately deformable flowing gel; “tongue” is clearly fixed
5	5.0	15:1	“F”—Highly deformable non-flowing gel; “tongue” is clearly fixed
6	5.0	100:1	“E”—Highly deformable flowing gel; “tongue” is weakly fixed
7	7.0	5:1	“H”—Slightly deformable non-flowing gel
8	7.0	10:1	“H”—Slightly deformable non-flowing gel; the “tongue” is not fixed—the gel is practically non-flowing
9	7.0	15:1	“G”—Moderately deformable flowing gel; “tongue” is clearly fixed
10	7.0	100:1	“F”—Highly deformable non-flowing gel; “tongue” is clearly fixed

**Table 3:** Stability of emulsions at different temperatures (after 8 h of observation).

N°	Dispersed Phase:Dispersion Medium Ratio	System Stability at Temperature, °C		
		25	40	70
1	80/20	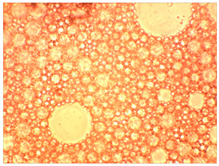 hydrocarbon phase separation < 0.5%	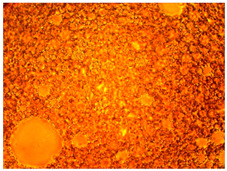 hydrocarbon phase separation < 3%	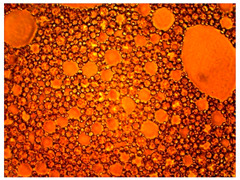 hydrocarbon phase separation < 5%
2	70/30	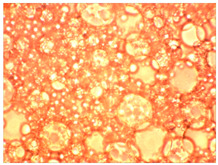 hydrocarbon phase separation < 5%	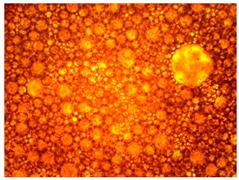 hydrocarbon phase separation < 5%	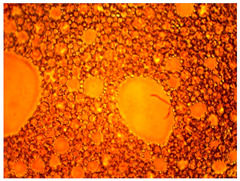 hydrocarbon phase separation > 5%
3	65/35	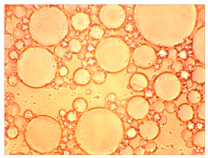 hydrocarbon phase separation > 5%The first signs of emulsion breakdown have appeared.	-	-

**Table 4:** Interaction of surfactant–polymer composition with formation fluids and displacement fluids (25 °C).

N°	Mixture Composition	Photo
1	Surfactant–polymer composition:fresh water = 1:1	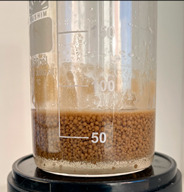
2	Surfactant–polymer composition:NaCl solution (ρ = 1.18 g/cm^3^) = 1:1	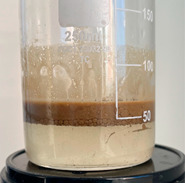
3	Surfactant–polymer composition:CaCl_2_ solution (ρ = 1.20 g/cm^3^) = 1:1	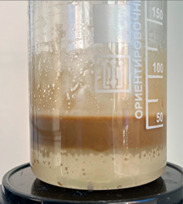
4	Surfactant–polymer composition: formation water of the Samara Region field (ρ = 1.17 g/cm^3^) = 1:1before filtration in the porous medium	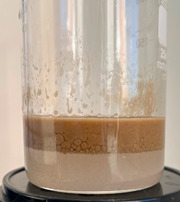
5	Surfactant–polymer composition:oil = 1:1	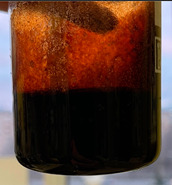

**Table 5:** Chemical composition of water.

Chemical Composition of Water, g/L	Value
Na^+^ + K^+^	76.8
Ca^2+^	11.5
Mg^2+^	3.4
Cl^−^	147.9
HCO_3_^−^	0.2
CO_3_^−2^	-
SO_4_^−2^	0.8
NH_4_^+^	-
Br^−^	-
J^−^	-
B^3+^	-
Li^+^	-
Sr^2+^	-
Rb^+^	-
Cs^+^	-
Total mineralization, g/L	240.8

**Table 6:** Sydansk classifier.

Code	Gel Structure
A	Initial polymer solution
B	Highly flowing gel
C	Flowing gel
D	Moderately flowing gel
E	Barely flowing gel
F	Highly deformable non-flowing gel
G	Moderately deformable flowing gel
H	Slightly deformable non-flowing gel
I	Rigid gel
J	«Ringing» Rigid gel

## Data Availability

The data presented in this study are openly available in the article.

## Abbreviations

OWC	Oil–water contact
HPAN	Hydrolyzed polyacrylonitrile
CA	Chromium acetate
$w_{pr0}^{w}$	initial water permeability at the experimental temperature
$w_{pr1}^{w}$	phase permeability to water
F_RR_	residual resistance factor
$w_{pr1}^{o}$	phase permeability coefficient for oil before exposure
$w_{pr2}^{o}$	phase permeability coefficient for oil after exposure
V_por_	pore volume
V_pump_	pumping volume
